# Evaluation of empathy among euthymic bipolar patients

**DOI:** 10.1192/j.eurpsy.2022.1056

**Published:** 2022-09-01

**Authors:** E. Mhiri, J. Ben Thabet, N. Smaoui, I. Gassara, R. Feki, L. Zouari, S. Omri, N. Charfi, M. Maalej, M. Maalej

**Affiliations:** Psychiatry C Department, Hedi Chaker, sfax, Tunisia

**Keywords:** Empathy, cognitive, affective, bipolar, euthymia

## Abstract

**Introduction:**

Bipolar disorder (BD) is a mental illness marked by extreme swings in the mood, energy, and thinking. Although it’s not an official symptom of the disease, some research suggests that it also may affect the empathy.

**Objectives:**

To investigate empathic responding in patients with BD in euthymic state of illness and to determine associated factors.

**Methods:**

A cross-sectional and descriptive study of 78 patients followed for bipolar disorder, during euthymia, at the psychiatric outpatient clinic at CHU Hédi Chaker in Sfax. We used a socio-demographic and clinical data sheet and the Questionnaire of Cognitive And Affective Empathy (QCAE) to assess empathy with its two dimensions : “Affective empathy” and “Cognitive empathy”.

**Results:**

The average age was 36.27 years, the sex ratio was 5.5. Bipolar I disorder was diagnosed in 88.5% of patients. The mean age of onset was 27.73 years, and the mean duration of illness was 8.4 years. 78.2% of patients had a good adherence to treatment. 60.3% of them had residual depressive symptoms during eutymia. QCAE total score was 72.49. (Maximum possible score 124) Cognitive empathy score was 43.21. (Maximum possible score 76) Affective empathy score was 29.36. (Maximum possible score 48) Affective empathy was associated with female gender (p=0), good adherence to treatment (p=0.01) and residual depressive symptoms (p=0.001).

**Conclusions:**

Our study shows that bipolar patients have fairly good levels of empathy. However, in order to better substantiate empathy in BD, comparative studies seem necessary.

**Disclosure:**

No significant relationships.

